# In Vitro and In Vivo Models for Drug Development Against Two Hemorrhagic *Hareavirales*: Rift Valley Fever and Crimean Congo Hemorrhagic Fever Viruses

**DOI:** 10.3390/v18030386

**Published:** 2026-03-19

**Authors:** Sarah Chaput, Antoine Nougairède, Franck Touret

**Affiliations:** Unité des Virus Émergents (UVE): Aix-Marseille Univ, Università di Corsica, IRD 190, Inserm 1207, IRBA, 13005 Marseille, France; chaputsarah@outlook.fr (S.C.); antoine.nougairede@univ-amu.fr (A.N.)

**Keywords:** Rift Valley fever virus, Crimean-Congo hemorrhagic fever virus, preclinical models, antivirals, hemorrhagic fever

## Abstract

Rift Valley fever virus (RVFV) and Crimean-Congo hemorrhagic fever virus (CCHFV) are designated by the World Health Organization as priority pathogens due to their epidemic potential, zoonotic transmission, and the absence of licensed therapeutics or vaccines. The development of effective antivirals critically relies on robust in vitro and in vivo models; however, progress is limited by the requirement for high-containment facilities. In this review, we provide a comprehensive overview of the experimental models currently available for RVFV and CCHFV, ranging from cell-based assays to animal models, and discuss their respective advantages, limitations, and translational relevance. We further highlight strategies allowing for BSL-2 experimentations, thereby expanding research accessibility, and accelerating the development of countermeasures against these high-priority pathogens.

## 1. Introduction

The emergence of viral diseases represents a major threat to public health, as illustrated by the recent SARS-CoV-2 pandemic, which resulted in substantial morbidity and mortality as well as profound socio-economic disruption worldwide. To improve preparedness for future epidemics, the World Health Organization (WHO) has published a global list of priority pathogens to guide the development and implementation of medical countermeasures [1]. Among these priority pathogens are members of the large viral families *Nairoviridae* and *Phenuiviridae*, within the order *Hareavirales* and the class *Bunyaviricetes* [2]. These families include highly pathogenic viruses affecting both humans and animals, notably Rift Valley fever virus (RVFV) and Crimean-Congo hemorrhagic fever virus (CCHFV).

Currently, no licensed therapeutics or vaccines exist for human use against RVFV or CCHFV [3,4], emphasizing the urgent need to pursue research in this area. Antiviral drug development is a lengthy, costly, and strictly regulated process, consisting of several key stages: discovery, preclinical testing, clinical trials, and regulatory approval. During the preclinical phase, candidate compounds are assessed both in vitro and in vivo for their efficacy, toxicity, and pharmacokinetic and pharmacodynamic properties. This phase critically depends on the use of appropriate study models, integrating both cell-based and animal models to efficiently screen candidates and characterize their properties before advancing them to clinical trials [5]. The availability of appropriate models remains a major challenge because work with these viruses requires high-containment biosafety level (BSL) laboratories (BSL-3 for RVFV and BSL-4 for CCHFV), due to their ability to cause severe outbreaks and their potential misuse as biological weapons. These laboratories require specialized infrastructure, multiple regulatory authorizations, and highly qualified personnel to operate, resulting in significant financial costs and posing major challenges for the research and development of such models.

This review introduces RVFV and CCHFV and provides an overview of available in vitro and in vivo models for antiviral development. We discuss their respective advantages, limitations, and translational relevance, and highlight solutions proposed by researchers to overcome challenges such as limited access to high-biosafety laboratories and the need for representative study models.

## 2. Rift Valley Fever Virus

### 2.1. General Aspects

RVFV is a member of the *Bunyaviricetes* class, *Phenuiviridae* family, and *Phlebovirus* genus. It is a mosquito-borne zoonotic arbovirus endemic to Africa, particularly East Africa, and has more recently been reported in the Arabian Peninsula. Since its identification in 1931 [6], RVFV has caused numerous outbreaks, the most recent in humans being reported in Senegal and Mauritania in 2025 [7].

Among animals, RVFV primarily infects domestic animals such as cattle, sheep, goats, and camels with sheep and goats being the most susceptible to severe disease. Infection in animals often results in high rates of abortion and mortality, leading to significant socioeconomic impact, particularly in sub-Saharan Africa, where livestock farming is a major source of income and nutrition. RVFV is primarily maintained in the environment through *Aedes* mosquitoes. These mosquitoes become infected either by feeding on infected animals or via vertical transmission to their eggs, which allows the virus to persist across mosquito generations during inter-epizootic periods. During outbreaks, other mosquitoes, such as *Culex*, also serve as horizontal vectors, amplifying transmission among animals and to humans. Environmental factors, particularly heavy rainfall, contribute to the transition from endemic maintenance to epizootic or epidemic outbreaks [8].

In humans, RVFV is transmitted through mosquito bites or by direct or indirect contact with blood or tissues of infected animals, placing individuals in close contact with livestock, such as farmers, veterinarians, and slaughterhouse workers, at higher risk [8]. Consumption of raw or unpasteurized animal products is also a risk of being infected with RVFV. Finally, vertical transmission in humans has been reported [9,10], but no human-to-human horizontal transmission. Most human cases are asymptomatic or present with mild flu-like symptoms; however, severe disease occurs in approximately 5% of symptomatic forms [4].

### 2.2. Virological Aspects

**Figure 1:**
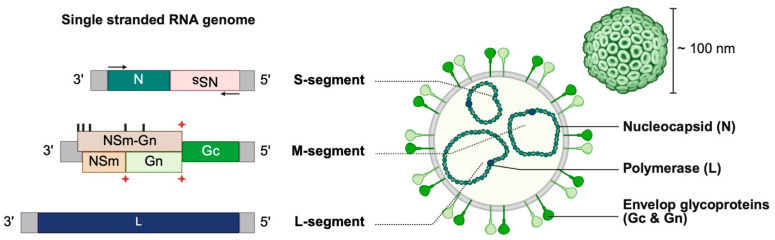
Structure and genomic organization of RVFV. The S segment has an ambisense organization, encoding the nucleoprotein N and the non-structural protein NSs. The M segment contains five alternative start codons (indicated by black symbols) that can be used for protein translation. Translation from the first start codon produces a polyprotein comprising the 78 kDa NSm-Gn (P78) and the glycoprotein Gc, while translation from the second start codon yields a polyprotein containing NSm, Gc, and Gn. These polyproteins are subsequently processed by cellular proteases at cleavage sites indicated by red symbols to generate the functional proteins. The L segment encodes the viral RNA-dependent RNA polymerase (L). Created in BioRender. Render4, B. (2026). BioRender.com/uqc5egw.

By electron microscopy, the RVFV virion is enveloped and exhibits a spherical or pleomorphic morphology with an average diameter of approximately 100 nm [11]. Its genome consists of three single-stranded RNA segments (Figure 1). The S-segment has an ambisense organization, encoding the nucleoprotein N in the antisense orientation and the non-structural protein NSs in the sense orientation. Through alternative start codons and co-translational cleavage, the M-segment, a negative-sense RNA segment, encodes the envelope glycoproteins Gn and Gc, as well as the non-structural proteins NSm (also known as P14) and NSm-Gn (or P78) [12]. Finally, the L-segment is a negative-sense RNA segment that encodes the RNA-dependent RNA polymerase L. Untranslated regions, at each segment termini, serve as promoters for transcription and replication by the viral polymerase, and facilitate segment circularization [13,14]. Circularized segments, together with N and L proteins, form ribonucleoprotein (RNP) complexes that are packaged into progeny virions via interactions with Gc [14,15].

The envelope glycoproteins Gn and Gc can adopt different conformations and allow the attachment of the virion to the host cell through interactions with diverse receptors [15]. These include C-type lectins, such as DC-SIGN and L-SIGN [16,17], heparan sulfate-based proteoglycans [18,19], and receptors from the low-density lipoprotein receptor family, such as LRP1 [20,21]. RVFV exhibits a broad cellular tropism, suggesting the involvement of other cellular factors remaining to be characterized [22]. Following attachment, entry occurs via a pH-dependent membrane fusion mediated by Gc, which functions as a class II fusion protein [22,23]. The nucleoprotein N encapsidates the viral genome, protecting it from degradation and immune recognition [24,25].

The non-structural proteins contribute to RVFV pathogenesis. NSs is a major virulence factor that inhibits type I interferon transcription, notably by forming a repressive complex on the interferon-beta (IFN-β) promoter [26,27,28,29]. NSs also seems to suppress host RNA transcription and prevents translational shutdown through multiple mechanisms, thereby facilitating viral protein synthesis [29]. NSm contributes by inhibiting apoptosis in infected cells [30]. Despite these insights, several aspects of the functions and mechanisms of RVFV non-structural proteins remain to be fully elucidated.

### 2.3. Human Clinical Features and Physiopathology

RVFV infection is typically mild or asymptomatic in most human cases, with an incubation period of 2 to 6 days. The disease typically presents as a non-specific viral syndrome, including fever, fatigue, myalgias, headache and chills, often accompanied by gastrointestinal symptoms such as nausea, vomiting, and diarrhea. Laboratory findings of infection typically include a usually moderate elevation of liver transaminases and lactate dehydrogenase (LDH) and leukoneutropenia. These symptoms generally resolve spontaneously within 4 to 7 days, although they may persist longer in some individuals [4,31,32,33,34,35]. RVFV exhibits broad cell tropism and can be detected in various organs including, but not limited to, the liver, brain, gastro-intestinal tract, kidneys, lungs, and spleen. Dendritic cells and macrophages are primary target cells, facilitating systemic viral dissemination once infected [36].

Approximately 5% of symptomatic cases develop complications, which are categorized into three severe syndromes: hemorrhagic fever (<1%), meningoencephalitis (<1%), and severe ocular manifestations (0.5–2%) [4,32]. The prevalence of these complications varies across outbreaks, and some patients may present with combination of these syndromes [4]. The severity of the symptoms is correlated with blood viral loads [37].

#### 2.3.1. Hepatitis and Hemorrhagic Fever

Hemorrhagic fever typically arises within the first few weeks, with lethality reaching up to 65% [4,34]. It is characterized by hemorrhagic signs including petechiae, gingival bleeding, hematemesis, and ecchymoses, and is invariably associated with thrombocytopenia and reduced hemoglobin levels. This syndrome is often accompanied by hepato-renal failure, disseminated intravascular coagulation (DIC) and/or encephalitis [4,31]. RVFV replicates early and extensively in the liver, causing significant damage including necrotic lesions [36,38]. Infection of hepatocytes and Kupffer cells has been demonstrated, suggesting that liver necrosis is directly induced by the virus [36]. Lesions are mainly localized in the mid-to-central zones of hepatic lobules but can also be more diffuse. Mild inflammatory infiltrates composed of lymphocytes and macrophages have also been reported [33,36]. Major hepatic involvement is reflected by highly elevated liver transaminases and LDH levels, with levels of elevation strongly correlated with severe disease. The resulting hepatocellular failure leads to a decreased synthesis of clotting factors [31,32,33,34,35].

#### 2.3.2. Meningo-Encephalitis

The meningoencephalitis form typically appears 1 to 4 weeks after the initial symptoms [4,32]. It can present as an acute, often fatal but short-lived form, or a subacute form, which is less severe but prolonged and frequently leaves sequelae [4]. The most common acute-phase symptoms include headache, neck stiffness, delirium, and retro-orbital pain, whereas the subacute form may involve dizziness, disorientation, and hallucinations [4,31,39,40,41,42,43]. Patients with this condition exhibit cerebrospinal fluid (CSF) enriched in proteins and white blood cells [35,39,41,43], as well as necrotic lesions and infiltration of lymphocytes and macrophages in the central nervous system (CNS) [39,43]. Neural cell infection has been demonstrated in animal models [44,45], but the mechanisms by which RVFV invades the nervous system remain incompletely understood [43]. Recently, Quellec et al. demonstrated in an in vitro human blood–brain barrier model that RVFV crosses the barrier via direct infection of the cells, without disrupting tight junction integrity, suggesting a mechanism of direct viral invasion [46].

#### 2.3.3. Ocular Form

The ocular form may lead to impairment or loss of central visual acuity, occurring from the onset of the disease up to several months later [31,47,48,49]. It typically develops 1 to 3 weeks after the initial symptoms, most commonly presenting with macular or paramacular edema [31,35]. Clinical manifestations include photophobia, reduced vision, scotomas, uveitis, retinitis, and retinal hemorrhages [31,35,47,48,49]. Many patients do not fully regain their vision, and scarring may persist [31,35,47,50]; however, partial improvement has been observed in some patients several months after infection [31,48,49]. The pathogenesis at the ocular level remains poorly understood [51].

#### 2.3.4. Immune Response

The human immune response to RVFV remains to be thoroughly characterized. With regard to adaptive immunity, seroepidemiological studies in endemic areas and follow-up of infected patients have demonstrated the presence of RVFV-specific antibodies [52,53,54]. High titers of neutralizing antibodies targeting the Gn glycoprotein indicate a post-infection humoral response directed against the virion surface glycoproteins [52,55].

Concerning innate immunity, RVFV inhibits the IFN-I antiviral response through its non-structural protein NSs, facilitating high viral loads that correlate with more severe disease [37,56]. Cytokine profiling in infected patients has revealed elevated levels of IL-10, an immunosuppressive cytokine [54,56,57]. However, findings regarding pro-inflammatory cytokines are inconsistent: one study associates them with survival [56], while another associates them with a fatal outcome [57]. The discrepancy between these findings is likely attributable to differences in the markers utilized or the number of samples examined. Regardless of the outcome of the disease, elevated levels of IL-6 and IL-8 have been observed in infected patients compared to uninfected individuals, indicating an inflammatory response [57]. Additionally, the chemokine CCL5, involved in leukocyte recruitment, is found at lower levels in fatal cases than in less severe infections [57].

Although the underlying mechanisms through which these immune factors influence disease progression remain unclear, current data suggest that, in addition to the inhibition of immune factors by the RVFV, dysregulation of the immune system may contribute to severe RVFV infection, while a well-regulated response may limit viral replication and reduce disease severity.

### 2.4. Virus Models: Strain, Engineered Virus, and Surrogate

#### 2.4.1. Wild-Type RVFV Strains

Most in vitro and in vivo efficacy studies use original clinical isolates and laboratory or recombinant strains. The ZH501 laboratory strain, also known as Zagazig Hospital 501, isolated during the 1977 outbreak in Egypt from a severe and fatal human case, is the most employed. Its widespread use facilitates comparison across different models, as viral virulence and pathology can vary by strain. However, these characteristics may also differ between viral batches, since ZH501 strain has been produced using various methods over time, likely selecting for distinct subpopulations [58]. Others used strains can be found in Table 1 and Table 2.

#### 2.4.2. Attenuated Viral Strains and Surrogates

Due to its high pathogenicity and the absence of licensed vaccines or treatments, RVFV must be handled under BSL-3 containment. In some countries, it is further classified as a select agent, requiring enhanced BSL-3 measures. Attenuated strains or surrogate viruses provide safer alternatives that can be used under BSL-2 conditions. Punta Toro virus (PTV), a related phlebovirus used as a RVFV surrogate, and the MP-12 strain have both been employed in in vitro and in vivo studies for the initial screening and characterization of antiviral compounds (Table 1 and Table 2) [74,75,76,77,100,101,103,115]. The MP-12 strain was generated through serial passage of the RVFV ZH548 strain in the presence of 5′-fluorouracil [116]. MP-12 carries 23 mutations throughout its genome [117], resulting in a significantly attenuated phenotype that permits its use under BSL-2 conditions, depending on national regulations.

#### 2.4.3. Engineered Viruses

The development of reverse genetics has led to the creation of tools such as fluorescent reporter strains, virus-like particles (VLPs) and pseudoviruses. Several of these systems have been used in vitro for the development of antiviral compounds against RVFV (Table 1).

Islam et al. replaced the NSs protein in ZH548 strain with the fluorescent protein Katushka to screen compound libraries and optimize the efficacy of antiviral candidates [59,60,61]. Using the same approach, other teams also employed the red fluorescent protein (RFP) [67] or luciferase [71] reporters. Keck et al. used the MP-12 strain, tagging the NSs gene with a Flag, to better understand the mechanism of action of Bortezomib while retaining all viral proteins [88]. In an effort to conserve viral proteins while using a wild-type strain (56/74), Nogales et al. recently developed and characterized a reporter RVFV by inserting the luciferase gene into the S-segment. They demonstrated that the insertion did not affect the viral kinetics in vitro and then conducted a proof-of-concept for an antiviral assay in vitro using ribavirin [91].

VLPs are non-infectious systems that can also be used depending on the experimental objective. Piper and Gerrard developed a minigenome system based on the ZH501 strain, enabling the production of T7 polymerase dependent VLPs upon transfection with plasmids expressing glycoproteins, N and L proteins in *trans*. This system incorporates a luciferase reporter for read-outs, allowing the evaluation of compounds such as ribavirin under safer conditions, including BSL-2 laboratories when permitted by national regulations [79]. Other non-replicative systems, such as those using S and L segments of the 35/74 strain with the GFP gene replacing NSs, similarly facilitate the study of antiviral dose–response [64,118].

Pseudoviruses can also be generated using the envelop proteins of other viruses, such as the vesicular stomatitis virus (VSV). Koehler et al. replaced the VSV glycoprotein gene with a luciferase gene and provided the RVFV glycoprotein in *trans*, enabling the testing of antiviral peptides targeting the glycoproteins [18,82]. Similarly, fluorescent VSV pseudoviruses incorporating RVFV glycoproteins have been used to study antiviral mechanisms, including fusion and cell attachment [90].

These engineered and surrogate systems are modular, facilitating compound screening and mechanistic studies, and in some cases allow BSL-3 bypass when non-replicative or non-infectious systems are used in accordance with national regulations.

### 2.5. In Vitro Models

In vitro cell models provide a first step for compound selection before their evaluation in in vivo models. They enable high-throughput screening, assessment of efficacy and toxicity, and elucidation of mechanisms of action. Table 1 summarizes the cell models used for antiviral development and evaluation against RVFV.

To date, the most used cell lines for RVFV are immortalized epithelial kidney cells derived from monkeys, including Vero and its derivatives, Vero 76 and Vero E6. Human cell lines are also used, including human embryonic kidney cells (HEK293) [70], pulmonary epithelial cells (A549) [59,60,61,62], skin-derived epithelial cells (Mel-JuSo) [64], retinal pigment epithelial cells (RPE) [67], osteosarcoma epithelial cells (U2OS) [68,90], hepatocellular carcinoma epithelial cells (Huh7.5) [92], and cervical carcinoma epithelial cells (HeLa) [80]. They are relatively easy to culture and cost-effective, with well-established techniques. However, they undergo genetic alterations, lack interactions with the extracellular matrix, and do not establish contact with other cell types, reducing their representativeness of in vivo conditions. Additionally, these cells may not accurately reflect the target tissue of replication in patients. Therefore, complementing assays in immortalized cells with primary cells and 3D models is recommended to improve the reliability of findings.

Primary cells can be used in antiviral development, and various cell types have already been tested for RVFV, including human small airway epithelial cells (HSAEC) [80,83,88,89], human brain microvascular endothelial cells (HBMEC) [68], and human umbilical vein endothelial cells (HUVEC) [84] (Table 1). Although primary cells are more expensive and have a limited lifespan, they are directly derived from donors and remain unmodified, making them more representative of in vivo responses. However, a key limitation is their availability, which varies depending on the tissue of origin [119]. These cells are typically cultured in two dimensions but can also be grown in three dimensions to generate more complex models, such as spheroids or organ-on-chip systems. Co-culture approaches are also feasible. For RVFV, one such model has been applied for antiviral testing: human primary hepatic spheroids composed of hepatocytes, endothelial cells, and Kupffer cells, available in 96-well plates [92] (Table 1). Additionally, the blood–brain barrier model employed to study RVFV pathogenesis in the brain could be adapted for drug development. This model employs a transwell system with immortalized human pericytes on the lower side, human endothelial brain-like endothelial cells derived from primary CD34^+^ human umbilical cord blood endothelial cells on the upper side, and commercially available human primary astrocytes within the well [46]. Beyond standard readouts, these complex systems also allow assessment of the innate immune response [46,120].

### 2.6. In Vivo Models

#### 2.6.1. Mice

Mice are widely used for studying RVFV pathogenesis and evaluating antiviral compounds (Table 2), offering advantages such as relatively low cost compared to other animal models. BALB/c mice are the most documented for RVFV infection, particularly for compound efficacy testing. In this model, RVFV causes severe disease with high lethality from 2 days post-infection (dpi) to 11 dpi following intraperitoneal (IP), subcutaneous (SC), or aerosol inoculation (Table 2). The disease initially presents with signs of fulminant hepatitis, including elevated liver enzymes and reduced albumin levels. Viral loads are high, particularly in the liver, but also in the kidneys, brain, and lungs. Mice succumbing later show encephalitic signs with neurological symptoms [44,62,66,93,94,95]. Histologically, lesions have been reported in the liver, intestines, and brain [44,62,66,93]. Overall, this model recapitulates severe human RVF, reflecting two severe manifestations: hepatitis, which may be associated with hemorrhagic fever, and later encephalitis. The ocular form is less characterized, but the virus has been detected in mouse eyes [44]. While this model is important due to its similarity to the severe human pathology, the disease is not uniform among individuals therefore requires the use of a larger number of animals.

Some mice strains exhibit only the hepatitis when infected with RVFV, including C57BL/6, 129Sv, and A/J [97,98]. Liver susceptibility appears to be partly determined by genetic factors, as specific genomic loci have been associated with the development of acute liver disease in MBT mice [121,122]. Upon SC or footpad (FP) exposure, C57BL6 mice are more susceptible than BALB/c, with total mortality before 4 dpi [97,98]. High viral loads are observed in the liver, spleen, blood and brain. Hematological and biochemical alterations, as well as liver and spleen damage, are comparable to those seen in BALB/c mice. With SC inoculation, no brain lesions or viral antigen are detected, likely due to early mortality [97].

To study neurological involvement, an encephalitis model was developed using the NSs-deleted ZH501 strain with intranasal (IN) inoculation in C57BL/6 mice. This strain does not cause overt disease when administered via SC or FP but induces encephalitic manifestation after IN exposure. However, its attenuated virulence may limit its utility for antiviral testing in certain contexts [123]. Using a wild-type strain, Cartwright et al. infected collaborative cross (CC) mice [99]. The CC panel is a genetically recombinant mouse population obtained through systematic crossbreeding of several strains, including five inbred and three outbred lines. The resulting mice were then inbred to generate mostly homozygous and genetically defined lines [124]. In their study, aimed at identifying a mouse model of RVFV-induced encephalitis, Cartwright et al. selected twenty CC strains carrying a functional wild-derived Mx1 locus, since the human homolog MxA has been shown to inhibit RVFV replication in vitro [99,125]. As a result, they demonstrated that CC057/Unc mice develop exclusively encephalitic manifestations when infected via FP injection [99].

To work under safer conditions and circumvent BSL-3 constraints, depending on national regulations, murine models using the attenuated MP-12 strain have been developed. This strain is non-virulent in immunocompetent mice, so immunodeficient IFNAR −/− [74] or STAT-1 KO [101] mice have been used (Table 2). These models lack efficient immune responses but allow initial compound screening under BSL-2 conditions before testing with virulent strains, reducing BSL-3-associated costs. Additionally, the surrogate Punta Toro virus (PTV) can be used in immunocompetent mice to evaluate antivirals targeting conserved proteins or functions shared by the two viruses [75,76,115]. Finally, the use of live-attenuated strains may also result in different phenotypes. Notably, in a study involving intranasal administration to immunocompetent BALB/c mice, the Clone 13 and Smithburn strains, used for livestock vaccination and obtained respectively through serial passages in mouse brains and through a natural deletion of the NSs protein, were associated with the development of encephalitis [96] (Table 2).

#### 2.6.2. Rats

Rats can also serve as models for developing RVFV countermeasures. Two comparative studies evaluated different strains of rats after SC inoculation or aerosols exposure [106], showing that clinical manifestations varied according to both strain and inoculation route (Table 2).

Several strains were highly susceptible to RVFV. Wistar and Brown Norway rats developed fulminant hepatitis with 100% lethality by 4 dpi following SC or aerosol infection [106,108]. In these rats, clinical symptoms, viral titers, and histological lesions closely resembled those observed in susceptible mice [44,97]. The Wistar model was notably employed to evaluate the antiviral efficacy of favipiravir [107]. In contrast, ACI and MAXX rats survived the first week after SC infection but subsequently developed clinical and histological signs of encephalitis, with high viral loads in brain and approximately 50% lethality between 11 and 16 dpi [108]. Aerosol infection in ACI rats accelerated disease progression, leading to more severe encephalitis, shortened survival to around 6 dpi and 100% lethality [106]. Therefore, this model of infection provides a more stringent encephalitis model, potentially useful for evaluating therapeutics.

Finally, other strains appeared more resistant to SC infection. This is the case for Lewis rats, which initially did not develop any notable clinical signs [108]. However, upon aerosol exposure, they exhibited 100% lethality with encephalitic manifestations similar to ACI rats [106], supporting their relevance as an encephalitis model (Table 2).

#### 2.6.3. Other Small Animals

Other small animal models have also been employed (Table 2). Subcutaneous RVFV infection in hamsters results in severe, fulminant hepatic disease with 100% mortality at 3–4 dpi, similar to that observed in C57BL6 mice. Post-mortem analyses revealed systemic viral dissemination, with severe hepatic and splenic involvement, and histopathological lesions resembling those observed in human and rodent hepatitis [36,38,97,106]. This model enabled antiviral testing of favipiravir, galidesivir, and ribavirin [73,81]. Additionally, it was also applied for BSL-2 studies, using the surrogate PTV [76,77], and the MP-12 attenuated strain in an immunodeficient STAT-2 KO hamster model [103] (Table 2).

The gerbil was also evaluated as a RVFV infection model via SC inoculation with encephalitis causing consistent mortality across inbred and outbred strains. However, age influenced susceptibility with three-week-old gerbils showing 100% lethality, while those over seven weeks had 61–90% survival [105]. Histopathological analyses revealed lesions similar to those reported in other animal models and in humans [39,43,44,105,106]. Therefore, young gerbils offer a relevant encephalitis model for antiviral evaluation, though reagent availability remains limited.

The ferret was recently investigated as a RVFV infection model, with IN inoculation inducing acute encephalitis. Although, they did not develop severe hepatitis, ferrets showed elevated liver enzymes and viral detection in multiple organs suggested systemic dissemination [104] (Table 2). Therefore, as a cost-effective, space-efficient, and non-rodent alternative to non-human primates (NHP), this model could complement rodent studies for assessing countermeasures targeting RVFV-induced encephalitis.

#### 2.6.4. Non-Human Primates

NHP models are critical for late-stage antiviral development due to their physiological similarity to humans. Various NHP models have been used for RVFV, as reviewed recently by Ebisine et al. [126] and are presented in Table 2.

The rhesus macaque has been the most widely used, exhibiting human-like RVFV pathology, with the most severe manifestations occurring particularly after intravenous (IV) inoculation [111,112,113,126]. Some macaques remain asymptomatic or develop a mild form of the disease, while others develop severe forms, with a fatality rate of 17%. Among the severe cases, two macaques died from hepatitis within the first week post-infection, while another succumbed later to acute encephalitis. The clinical symptoms and histopathological lesions observed in the liver and brain closely resemble those reported in humans and other animal models [36,39,111]. Furthermore, hemorrhagic manifestations, such as petechiae and DIC, have been reported [111].

Other NHP species have also been evaluated as models for RVFV infection. Smith et al. evaluated the common marmoset by testing several routes of exposure (IV, SC and IN). Marmosets proved more sensitive than rhesus macaques, exhibiting severe clinical signs, viremia, and more pronounced biochemical and hematological abnormalities. Pathology was route-dependent with IV exposure inducing a hemorrhagic form, characterized by 75% lethality, DIC, thrombocytopenia, and fibrin thrombi; SC exposure resulting in either hepatitis or late-stage encephalitis with 50% mortality; and IN infection leading to uniformly lethal late-stage encephalitis and interstitial pneumonia. Notably, all animals infected via the IN route showed viral presence in retina, suggesting this model could also be valuable for studying ocular manifestations. A subsequent study confirmed marmoset sensitivity, with infection via aerosols. The hematological changes were comparable to those following IN inoculation, with the addition of reduction in platelet count and changes in platelet size, suggesting platelet turnover and a thrombotic process. Encephalitic forms were only observed at the highest doses and in animals that succumbed to the infection [114]. Therefore, marmosets offer a model of severe disease with tunable clinical presentations based on inoculation route.

The African green monkeys (AGM) were also found to be susceptible to aerosol infection, although at higher doses than marmosets, with 5 of 6 animals succumbing to encephalitis [114]. Hematological parameters in AGMs were comparable to those in marmosets, although no elevation in liver enzymes (ALT and ALP) was observed, suggesting limited hepatic involvement [114]. Aerosol-infected AGMs therefore constitute another relevant NHP model for studying the encephalitic forms of RVFV and evaluating antiviral countermeasures.

## 3. Crimean Congo Hemorrhagic Fever Virus

### 3.1. General Aspects

CCHFV belongs to the class *Bunyaviricetes*, family *Nairoviridae* and genus *Orthonairovirus*. It was identified in 1969 as the etiological agent of both Crimean fever (first described in 1944) and Congo fever (1956) [127,128]. It is a globally distributed tick-borne virus, present in Africa, the Middle East, Southern Asia, and Southern and Eastern Europe. Its distribution closely follows that of its primary vector, *Hyalomma* spp. ticks.

CCHFV is the arbovirus with the highest known genetic diversity. Multiple genotypes can be distinguished, each with a characteristic geographical distribution [129]. Traditionally, seven genotypes have been recognized: Africa 1 to 3, Asia 1 and 2, and Europe 1 and 2 [130]. However, the International Committee on taxonomy of viruses (ICTV) has recommended reclassifying clade Europe 2 as a distinct species within the genus *Orthonairovirus,* now designated *Orthonairovirus parahaemorrhagiae* (Aigai virus) [2,131].

Humans are most commonly infected with CCHFV through tick bites, but infection can also occur via direct contact with infected livestock or direct exposure to infected blood, organs, or body fluids [3]. A wide range of wild and domestic animals serve as amplifying hosts, supporting viral transmission between ticks; however, humans are the only species that may develop symptoms [3]. In humans, CCHFV is responsible for severe hemorrhagic fever, with a case-fatality rate ranging from 5 to 30% depending on studies and region [3,132].

### 3.2. Virological Aspects

**Figure 2:**
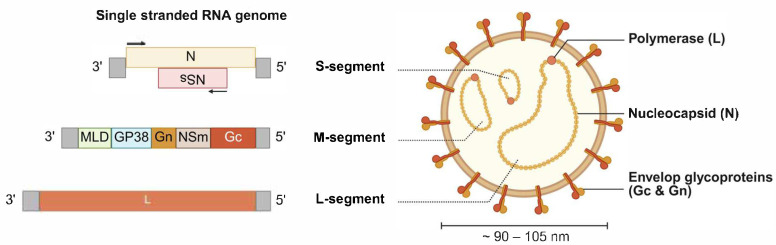
Structure and genomic organization of CCHFV. Schematic representation of the CCHFV virion and its genome organization. The S segment has an ambisense organization and encodes the nucleoprotein N and the non-structural protein NSs. The M segment encodes the precursor GPC that will be proteolytically processed to produce the envelope glycoproteins Gn and Gc, the non-structural protein NSm, the glycoprotein GP38, and a mucin-like domain (MLD). The L segment encodes the viral RNA-dependent RNA polymerase (L). Created in BioRender. Render4, B. (2026). BioRender.com/z55r13g.

When observed by electron microscopy, CCHFV particles display a spherical morphology with a diameter of 90 to 105 nm and surface projections of 8 to 10 nm [133]. The viral genome consists of three single-stranded RNA segments (Figure 2). The S segment encodes the nucleoprotein N in the antisense orientation and the non-structural protein NSs in the sense orientation [134,135]. The M segment encodes in the negative sense the nonstructural protein NSm, the envelope glycoproteins Gn and Gc, the nonstructural glycoprotein GP38, and a mucin-like domain (MLD) [3,136,137,138]. The L segment encodes in the negative sense the RNA-dependent RNA polymerase (L protein) [3,139].

As in RVFV, the untranslated regions at both termini of each segment contain signals required for transcription, replication, and packaging into nascent virions [14,140,141]. The nucleoprotein N protects viral RNA and drives formation of ribonucleoprotein (RNP) complexes that interact with glycoproteins to ensure encapsidation [142,143,144]. By binding the 5′ untranslated region and host ribosomes, N enhances translation of viral mRNAs over cellular mRNAs [145]. It also exhibits in vitro endonuclease activity of unclear function [146] and contains a conserved caspase-3 cleavage site, which may regulate RNA synthesis and apoptosis [142,147,148].

Gc and Gn mediate receptor binding, membrane fusion, and viral entry, a process dependent on low pH and clathrin-mediated endocytosis [149]. Identified host receptors include the low-density lipoprotein receptor (LDL-R) [150,151,152], the C-type lectin DC-SIGN [153], and potentially nucleolin [154]. The functions of MLD and GP38 remain incompletely understood. When still bound to GP38, MLD seems to regulate Golgi accumulation of glycoproteins, reducing particle numbers while enriching envelope glycoproteins, whereas GP38 is essential for infectious particle formation by enabling Gn/Gc maturation and incorporation [136].

The role of NSm is not fully elucidated. It appears to facilitate Gc maturation and the release of infectious particles [136], although it is dispensable for replication and virulence in interferon receptor-deficient (IFNAR −/−) mice [155]. Nevertheless, NSm-deficient strains show slower replication kinetics in interferon-competent cells compared with interferon-deficient cells [155]. Moreover, in a CCHFV strain adapted to an immunocompetent mouse model, a nonsynonymous mutation emerged within the NSm coding region, supporting a role in viral adaptation to the murine host and possibly in the evasion of the murine innate immune response [156]. In contrast, NSs is critical for modulating apoptosis to favor replication, either by triggering caspase activation or disrupting mitochondrial membrane potential, though the precise mechanisms remain unclear [135].

Finally, the viral polymerase provides RNA-dependent RNA polymerase activity, mediates “cap-snatching” and possesses an ovarian tumor-like (OTU) protease domain which blocks the type I interferon antiviral response mediated by RIG-I [3,157].

### 3.3. Human Clinical Features and Physiopathology

#### 3.3.1. Crimean-Congo Fever

Clinical manifestations of CCHFV infection range from asymptomatic or mild illness to a severe disease, named Crimean-Congo hemorrhagic fever (CCHF). Reported case-fatality rates average around 30%, but vary widely depending on study size, geographic region, and time period, as reviewed by Nasirian [132]. Importantly, underdiagnosis of mild or asymptomatic cases leads to overestimation of the true case-fatality rates.

CCHF typically progresses through four phases: incubation, pre-hemorrhagic, hemorrhagic, and convalescent. The incubation period usually ranges from 1 to 9 days, being shorter following tick bites than after contact with infected blood or tissues [3], although prolonged incubation up to 53 days has been reported [158]. The pre-hemorrhagic phase typically lasts 2 to 5 days and is characterized by nonspecific symptoms including fever, headache, myalgia, nausea, and vomiting. The subsequent hemorrhagic phase generally lasts 2 to 3 days but can extend up to 2 weeks. This stage is marked by bleeding manifestations, such as petechiae, ecchymoses, and mucosal hemorrhages (epistaxis, hematemesis, melena, and hematuria). Laboratory findings during CCHF typically include elevated liver transaminases, increased LDH levels, and leukoneutropenia, sometimes associated with renal failure and hepatocellular insufficiency. Hemorrhagic symptoms are particularly pronounced in CCHF compared with some other viral hemorrhagic fevers, such as RVF. Severe hepatic and renal dysfunction are common, and in critical cases, disseminated intravascular coagulation, shock, and multi-organ failure may occur [3,159,160,161].

The convalescent phase usually begins between days 9 and 20 post-infection, with gradual normalization of laboratory parameters. However, full recovery may require several months, during which patients frequently experience fatigue, memory impairment, alopecia, cardiovascular abnormalities (hypotension, tachycardia, or bradycardia), and sensory disturbances [3,159,160,161].

Several clinical and laboratory markers have been associated with disease severity, including high viral load [162,163,164], thrombocytopenia, elevated levels of liver transaminases, LDH, and creatine phosphokinase levels, as well as prolonged clotting time [161,165,166,167].

#### 3.3.2. Pathogenesis and Immune Response

Immune cells appear to be early targets of CCHFV infection, particularly monocytes, activated macrophages, and dendritic cells (reviewed in [168]). The innate immune response represents the first line of defense, primarily mediated by type I interferon response, which restricts viral dissemination [3,168]. Its protective role has been demonstrated both in vitro [169] and in vivo, as inactivation of the interferon pathway in mice results in rapid fatality, whereas immunocompetent mice remain asymptomatic [170,171,172]. In humans, certain polymorphisms in toll-like receptor genes, involved in viral RNA sensing and interferon induction, are associated with increased disease severity [173,174]. CCHFV has evolved several immune evasion strategies, notably through the OTU protease domain of the L protein and via the apoptosis regulation mediated by NSs and N proteins [135,142,147,148,157]. Dysregulation of the immune response, characterized by excessive production of pro-inflammatory cytokines such as MCP-1, IL-6, and IL-8, is associated with severe disease [175].

The adaptive immune response contributes to viral control. IgM antibodies arise rapidly after infection and then disappear within a few months, whereas IgG antibodies appear later and may persist for years [176]. Early responses predominantly target the N protein, followed by glycoproteins. The presence of anti-N IgM correlates with improved viral clearance [177], and overall antibody production is associated with survival, as antibodies are often undetectable in fatal cases [168,176]. A temporal association between IgM/IgG emergence and viremia decline further supports their role in infection control [168,176]. Nonetheless, antibody titers and neutralizing capacity have not been clearly linked to disease severity in murine or primate models [178,179]. In humans, neutralizing antibodies are rare and usually detected at low levels in convalescent individuals, suggesting they are not essential for recovery [176]. Thus, the precise contribution of humoral immunity to protection against CCHFV remains uncertain.

T-cell responses also play a critical role. While early activation and strong inflammatory responses correlate with severe disease [162,167], both CD4^+^ and CD8^+^ T cells are required for viral control in IFN-I-deficient mouse models [179]. In humans, antigen-specific CD8^+^ memory responses, primarily directed against the nucleoprotein, can persist for over a decade post-infection [180]. As with antibody, the balance between protective T-cell activity and immunopathology appears critical in determining clinical outcome [168].

### 3.4. Virus Models: Strain, Engineered Virus, and Surrogate

#### 3.4.1. Wild-Type CCHFV Strains and Surrogates

The IbAr 10200 laboratory strain, isolated from a tick in 1966 in Nigeria, is the most widely used reference for in vitro and in vivo CCHFV research (Table 3 and Table 4). This strain is valuable because of its widespread use, and its extensive characterization facilitates reproducibility and comparison of experimental findings. However, its human pathogenicity remains unclear [181,182] and studies have highlighted its divergence from pathogenic strains currently circulating in humans [183]. In addition, multiple laboratory passages, including in newborn mice brain, may have introduced adaptations to culture models further increasing its differences from circulating strains. Therefore, inclusion of strains circulating in humans is essential as strain-specific differences in antiviral susceptibility can affect the outcomes. Several clinical isolates from human cases that are more recent, with minimal passaging, and no adaptation in mice, have also been employed both in vitro and in vivo, including Kosova Hoti [184], Afg-09 2990 [185], and YL16070 (GenBank KY354082) (Table 3 and Table 4).

#### 3.4.2. BSL-2 Surrogate and Engineered Viruses

A major challenge in studying CCHFV is the requirement for BSL-4 facilities. As with RVFV, several surrogate systems have been developed to enable research under BSL-2 conditions, which are more accessible.

The Hazara virus (HAZV), an *Orthonairovirus* closely related to CCHFV, serves as one such surrogate model. HAZV can be used in BSL-2 laboratories as a fully infectious virus to evaluate therapeutic candidates in vitro and in vivo [188,189,197,213] (Table 3). This model has been applied to characterize different antiviral compounds [189,197], as well as to validate screening results and dose–response data obtained with CCHFV and HAZV minigenome systems [188]. Furthermore, an in vivo mouse model for HAZV infection has also been established and will be discussed later in this review [213].

Minigenome systems provide a powerful, non-infectious tool for assessing antiviral activity against CCHFV under BSL-2 conditions (Table 3). Hirano et al. developed a CCHFV minigenome by co-transfecting plasmids encoding the viral L and N proteins together with a construction containing the 3′ and 5′ ends from the L segment with a reporter (luciferase). This system enabled high-throughput compound screening and dose–response evaluation [188]. Similarly, Liu et al. employed a GFP-based minigenome to elucidate the mechanism of action of baloxavir against CCHFV [198].

Finally, pseudotyped viruses have been used in vitro to assess potential CCHFV inhibitors targeting viral entry and fusion (Table 3) [183,186,192]. These pseudoviruses are typically based on VSV envelop carrying CCHFV glycoproteins, and engineered to express a reporter gene, such as GFP [186] or luciferase [183,192]. Notably, Zivcec et al. (2017) [192] demonstrated that pseudoviruses enable the evaluation of cross-neutralization activity of antibodies against multiple CCHFV strains by expressing strain-specific glycoproteins, thereby circumventing the need to manipulate all these infectious viral strains in BSL-4 facilities.

### 3.5. In Vitro Models

Immortalized cell lines are widely used to assess antiviral compounds against CCHFV. In vitro, SW-13 cells, derived from human adrenal cortical carcinoma, are frequently employed [186,187,188,192,193]. Other human cell lines used include HEK293 kidney cells [199], A549 lung epithelial cells [194,197], and Huh7.5 hepatoma cells [186,187,195] (Table 3). Immortalized cell lines from other animal species, commonly used in virology, have also been applied in CCHFV research, including Vero E6 cells, derived from African green monkey kidney epithelium [189,191,195,196], and BHK-21 cells, originating from newborn hamster kidney fibroblasts [186,188] (Table 3). While these cell lines are useful, complementary models that better recapitulate human physiology are valuable for validating and refining results.

Few studies have employed primary cells or more complex in vitro models to evaluate antiviral compounds against CCHFV. Müller et al. used primary human hepatocytes to determine dose–response relationships and cytotoxicity for CR-31-B (-) and silvestrol against CCHFV and Lassa virus (*Arenaviridae*), another hemorrhagic fever virus [190]. Liu et al. assessed baloxavir in vitro using primary human umbilical vein endothelial cells (HUVEC), establishing dose–response curves, cytotoxicity and investigating its mechanism of action via time-of-addition experiments [198]. Additionally, primary human macrophages were used to compare the infectivity of VLPs bearing glycoproteins from different CCHFV strains [183].

To date, organotypic models have not been applied for CCHFV antiviral development. However, in recent a study identifying the low-density lipoprotein receptor (LDLR) as a receptor for CCHFV attachment and entry, findings were confirmed using blood vessel organoids [152]. Such organoid model could be explored to validate the activity of antiviral compounds initially identified in immortalized or primary cells, providing a more physiologically relevant context prior to in vivo experimentation.

### 3.6. In Vivo Models

Progress in understanding CCHFV infection has been limited by the lack of suitable animal models. For many years, only newborn mice were available for evaluating antiviral strategies. However, their immature immune system restricted their utility for such studies. It was not until 2010 that more relevant alternative models were developed. These models are summarized in Table 4.

#### 3.6.1. Mice

Several immunodeficient mouse models have been employed to study CCHFV infection, including C57BL6 IFNAR −/−, 129Sv IFNAR −/− et 129S6/Sv IFNAR −/−, and C57BL6 mice transiently immunocompromised with anti-IFNAR antibodies (Table 4). All these models develop severe disease resembling human CCHF, with marked liver and spleen damage. The most affected cell types include hepatocytes, endothelial cells, and macrophages, including Kupffer cells [170,172,191,196,214]. Hematological abnormalities, such as thrombocytopenia, prolonged coagulation times, and elevated liver enzymes are reported, consistently with disease markers of severity in humans. Leukopenia is also observed in mice and frequently reported in human CCHF cases [161,165,166,167,170,172]. Cytokine analyses reveal a pronounced pro-inflammatory response, similar to that in patients [167,170,172,196,215]. Various inoculation routes have been used, including intraperitoneal, subcutaneous, and intradermal injections (Table 4). The uniform lethality within 2 to 7 days, combined with the severity and similarity to human pathology, makes these models valuable for antiviral testing, despite limitations due to immunosuppression.

A humanized female mouse model has also been described [208] (Table 4). These mice were infected with two CCHFV strains, of which only the Turkey-200406546 strain caused uniformly lethal infection, with death occurring between 12 and 23 days post-infection. Liver and spleen pathology resembled that in immunodeficient mice, although only rare hepatocytes were positive for viral antigen. Notably, brain involvement was also observed, a feature not reported in other models, suggesting different viral tropism. High viral titers and viral antigen were detected in astrocytes, glial cells, and some neurons [208]. Rare neurological symptoms have been reported in humans [216,217], suggesting this model could be useful for studying CCHFV human neurotropism and neuropathogenicity. However, the availability of other mouse models that recapitulate human disease limits the utility of humanized mice for antiviral development, given the practical constraints of using humanized mice.

In general, immunocompetent mice do not develop diseases following CCHFV infection. However, Hawman et al. (2021) reported an immunocompetent mouse model by adapting the Hoti strain through eleven passages in the mouse liver [156]. The resulting strain, MA-CCHFV, caused lethal infection in 83% of C57BL6 mice at 10^5^ TCID_50_ [207] (Table 4). A marked sex-dependent difference in susceptibility was observed with female exhibited only mild disease, whereas males developed a severe form resembling that seen in immunodeficient mice, although survival varied depending on the inoculated dose [156,207]. The basis for this sex difference remains unexplained, and the adapted strain does not correspond to circulating strains. Nevertheless, this model is unique in reproducing severe disease in immunocompetent rodents and provides a valuable alternative to immunodeficient models. Male mice from this model have notably been used to evaluate the efficacy of several antiviral compounds against CCHFV [207] (Table 4).

A BSL-2 surrogate model has also been described, using 129Sv IFNAR −/− mice infected with HAZV. This model produces disease with characteristics similar to human CCHF, including liver and spleen pathology. The disease is uniformly lethal within 5 days depending on the inoculated dose of virus (ID inoculation) and presents a pathology very similar to CCHFV in immunodeficient mice. It includes significant weight loss, viral titers detected in the liver, spleen, and lymph nodes, and histological changes noted in the same organs [213]. These similarities make this model a useful surrogate for BSL-4 studies and evaluation of antiviral compounds.

#### 3.6.2. Other Small Animals

To date, only one small animal model other than mice has been described for CCHFV therapeutics development: the STAT2 −/− Syrian golden hamster [209] (Table 4). SC infection in this model is uniformly lethal, and animals develop pathology similar to that seen in immunodeficient mice and humans, including liver and spleen lesions, elevated liver enzymes, and prolonged coagulation times [209]. However, some differences are observed, notably an increase in white blood cell counts and the absence of IL-6 or IL-8 upregulation, cytokines associated with disease severity in humans [175]. Furthermore, some infected hamsters exhibit hemorrhagic signs, including petechiae, which are absent in immunodeficient mouse models but reflect features of human CCHF pathology [3,209]. Therefore, this model could serve as a useful alternative to mouse models for therapeutic development.

#### 3.6.3. Non-Human Primates

Rhesus macaques and African green monkeys have been evaluated as potential models for CCHFV infection, but infection in these species did not result in overt disease [178]. In contrast, cynomolgus macaques develop moderate to severe illness, with some animals succumbing to infection [178,210,211]. In the initial characterization of this model, Haddock et al. tested several exposure routes (SC, IV, and combined SC + IV). SC inoculation produced only mild disease, whereas IV and SC + IV routes resulted in more severe disease, with the highest mortality following IV administration, although pathology was similar between the two routes [178]. Mortality, when observed, generally occurred between 5 and 7 days after infection [178,210].

Subsequent studies reported variable outcomes reflecting inter-individual variability. Cross et al. observed only mild disease and 100% survival [211]. In the most severe cases, clinical signs included anorexia, dehydration, edema, gastrointestinal hemorrhage, petechiae, and internal bleeding. Hematological and biochemical analyses revealed leukopenia, thrombocytopenia, elevated liver enzymes, and increased inflammatory cytokines, consistent with human severe CCHF [178,210]. Histopathological lesions were primarily observed in the liver and spleen, with viral antigen detected in high amounts in blood, lymph nodes, liver, spleen, adrenal glands, and kidneys [178]. Although this model is not uniformly lethal and presents a spectrum of outcomes, necessitating larger animal cohorts, it remains the most relevant NHP model currently available and has been successfully used to evaluate antiviral efficacy, including favipiravir [210].

To enhance lethality, Hawman et al. (2024) adapted the Hoti strain of CCHFV through four serial passages in the liver of cynomolgus macaques [212]. Due to restricted access to cynomolgus macaques during the SARS-CoV-2 pandemic, the adapted strain was subsequently tested in rhesus macaques [212], a species typically resistant to wild-type strains [178]. Combined IV and SC inoculation resulted in viremia in all animals, with some exhibiting thrombocytopenia and elevated liver enzymes. Viral RNA was primarily detected in lymph nodes, liver, and spleen of animals exhibiting the most pronounced biochemical alterations [212]. Although the adapted strain induced only mild to moderate disease, this model offers a useful alternative for therapeutic evaluation, particularly when cynomolgus macaques are not available.

## 4. Conclusions

RVFV and CCHFV are two WHO-priority pathogens of particular concern due to their epidemic potential, their impact on humans and animals, and the lack of licensed treatments or vaccines. The development of antivirals relies critically on suitable in vitro and in vivo models. In this review, we present the currently available models for these two viruses, including approaches that enable initial evaluation under BSL-2 conditions, followed by confirmation with the pathogens of interest.

For both viruses, primary cell or organotypic models remain limited. Nonetheless, their physiological relevance and potential to reduce animal experimentation suggest an increasing role in the development of new therapies. Regarding animal models, numerous models are available for RVFV, but the diversity of clinical presentations in humans requires the use of complementary models. For CCHFV, the absence of uniformly severe disease in immunocompetent animals, particularly non-human primates, complicates antiviral evaluation. Despite this, the immunodeficient mouse models and the cynomolgus macaque model remain highly valuable and closely recapitulate human disease.

Finally, the choice of viral strains is also crucial. Laboratory strains facilitate standardization, but testing candidates against recent, low-passage isolates is essential to ensure translatability of results.

In conclusion, improving and diversifying preclinical models, alongside judicious use of the available approaches, is essential to accelerate the development of effective therapeutic strategies against RVFV and CCHFV. For these viruses, as well as for other emerging pathogens, sustained research efforts during inter-epidemic periods are crucial to maintain robust and validated tools that will enable rapid and effective responses to future outbreaks.

## Figures and Tables

**Table 1:** In vitro antiviral studies against RVFV or surrogate PTV.

Virus (Strain)	Specificity	Cell Line (Type and Origin)	Assays (Readout)	References
RVFV (ZH548 ΔNSs-Katushka)	Reporter virus (fluorescent)	A549 (Human lung epithelial cells; I)	Screening, dose response, and MOA (fluorescence); toxicity (resazurin reduction)	Islam et al. 2016 [59], 2018 [60], 2022 [61]
RVFV (ZH501 and MP-12)	-	Vero (African green monkey epithelial kidney cells; I); A549 (Human lung epithelial cells; I)	Dose response and MOA (infectious titer); toxicity (cell staining)	Johnson et al., 2022 [62]
RVFV (AR20368)	-	Vero (African green monkey epithelial kidney cells; I)	Screening (Infectious titer); toxicity (MTT reduction)	More et al., 2021 [63]
RVFV (35/74)	VLP expressing a fluorescent protein	Mel-JuSo (Human cutaneous epithelial cells; I)	Dose response (fluorescence); toxicity (resazurin and WST-1 reduction, cell staining)	Luteihn et al., 2020 [64]
RVFV (ZH501)	-	Vero (African green monkey epithelial kidney cells; I)	Dose response (virus detection by cell-based ELISA); toxicity (ATP quantification)	Saikh et al., 2020 [65]
RVFV (35/74 and MP-12)	-	Vero76 (African green monkey epithelial kidney cells; I)	Dose response (virus neutralization)	Gutjahr et al., 2020 [66]
RVFV (ZH548 ΔNSs-RFP)	Reporter virus (fluorescent)	RPE (Human retinal pigment epithelial cells; I)	Dose response (fluorescence); toxicity (ATP quantification)	Andersen et al., 2019 [67]
RVFV (MP-12)	-	U2OS (Human-derived osteosarcoma epithelial cells; I); HBMEC (Human brain microvascular endothelial cells; P)	Dose response (viral RNA and infectious titer); toxicity (cell count)	Hackett et al., 2019 [68]
RVFV (56/74)	-	Vero (African green monkey epithelial kidney cells; I)	Dose response and resistance barrier (infectious titer)	Borrego et al., 2019 [69]
RVFV (MP-12)	-	HEK 293 (Human embryonic kidney cells; I)	Dose response and MOA (infectious titer); toxicity (resazurin reduction)	Ellenbecker et al., 2014 [70]
RVFV (MP-12 ΔNSs-Luc)	Reporter virus (luminescent)	H2.35 (Mouse liver hepatocyte cells; I)	Dose response (luminescence, infectious titers); toxicity (ATP quantification)	Bell et al., 2018 [71]
RVFV (cell-culture-adapted)	-	Vero (African green monkey epithelial kidney cells; I)	Dose response (viral RNA, infectious titer); toxicity (MTT reduction)	Ahmed et al., 2023 [72]
RVFV (ZH501)	-	Vero76 (African green monkey epithelial kidney cells; I)	Dose response (infectious titer); toxicity (cell staining)	Westover et al., 2018 [73]
RVFV (MP-12)	-	Vero76 (African green monkey epithelial kidney cells; I)	Dose response (infectious titer); toxicity (cell staining)	Smee et al., 2018 [74]
PTV (Adames, Balliet)	-	LLC-MK_2_ (Monkey Rhesus epithelial kidney cells; I); MA-104 (African green monkey epithelial kidney cells; I); Vero (African green monkey epithelial kidney cells; I)	Dose response (infectious titer); toxicity (cell staining)	Sidwell et al., 1988 [75]
PTV (Adames)	-	Vero76 (African green monkey epithelial kidney cells; I)	Dose response (infectious titer); toxicity (cell staining)	Gowen et al., 2007 [76]
RVFV (MP-12), PTV (Adames)	-	Vero76 (African green monkey epithelial kidney cells; I)	Dose response (infectious titer); toxicity (ATP quantification)	Gowen et al., 2008 [77]
RVFV (MP-12)	-	Vero76 (African green monkey epithelial kidney cells; I)	Dose response (infectious titer); toxicity (cell staining)	Selvam et al., 2007 [78]
RVFV (ZH501 VLP ΔNSs-luciferase and MP-12)	Reporter virus (luminescent)	Not indicated	Dose response (immunofluorescence (N protein; MP-12) or Luciferase (VLP))	Piper and Gerrard, 2010 [79]
RVFV (MP-12 and ZH501)	-	HeLA (Human uterine epithelial cells; I); HSAEC (Human small airway epithelial cell; P)	Screening, dose response, and MOA (immunofluorescence (Gn protein)); toxicity (cell count)	Mudhasani et al., 2014 [80]
RVFV (ZH501)	-	Vero76 (African green monkey epithelial kidney cells; I)	Dose response (infectious titer); toxicity (cell staining)	Scharton et al., 2014 [81]
RVFV (ZH501, MP-12) VSV-luc (ΔNSs-luciferase, RVFV-G)	Pseudovirus expressing a luminescent protein	VeroE6 (African green monkey epithelial kidney cells; I)	Screening, dose response, and MOA (infectious titer, viral RNA, luminescence); toxicity (MTT reduction)	Koehler et al., 2013 [82]
RVFV (MP-12, ZH501)	-	HSAEC (Human small airway epithelial cells; P); A549 (Human lung epithelial cells; I)	Screening (infectious titer); toxicity (ATP quantification)	Narayanan et al., 2012 [83]
RVFV (BJ01)	-	HUVEC (Human umbilical vein endothelial cells; P)	Dose response and MOA (viral RNA, N protein quantification); toxicity (WST-8 reduction)	Li et al., 2021 [84]
RVFV (MP-12)	-	Not indicated	Dose response (infectious titer); toxicity (cell staining)	Kryshchyshyn-Dylevych et al., 2023 [85]
RVFV (MP-12)	-	Vero (African green monkey epithelial kidney cells; I)	Dose response (immunofluorescence (N protein)); toxicity (WST-1 reduction)	Shimojima et al., 2014 [86]
RVFV (MP-12)	-	Vero (African green monkey epithelial kidney cells; I)	Screening, dose response, and MOA (infectious titer); toxicity (MTT reduction)	Alkan et al., 2024 [87]
RVFV (MP-12, Flag-NSs-MP-12 virus)	Tagged virus	HSAEC (Human small airway epithelial cells; P)	Dose response and MOA (infectious titer); toxicity (ATP quantification)	Keck et al., 2015 [88]
RVFV (MP-12, ZH501)	-	HSAEC (Human small airway epithelial cells; P)	MOA (infectious titer); toxicity (ATP quantification)	Anderson et al., 2023 [89]
RVFV (MP-12, VSV-RVFV-GFP, ZH501)	Pseudovirus expressing a fluorescent protein	Cortical neurons (Rat E18 Sprague Dawley IGS, C57BL6 and C57BL6 TRL2 KO mice; P); U2OS (Human-derived osteosarcoma epithelial cells; I)	Screening, dose response, and MOA (infectious titer, immunofluorescence (Gn protein), viral RNA)	Griesman et al., 2024 [90]
RVFV (S-luc-56/74)	Reporter virus (luminescent)	VeroE6 (African green monkey epithelial kidney cells; I)	Dose response (luminescence)	Nogales et al., 2024 [91]
RVFV (Chad 2001 and H13/96)	-	Huh7.5 (Human liver epithelial cells; I); Liver spheroid (Human hepatic cells: hepatocytes, Kupffer and endothelial cells; P)	Dose response (viral RNA, infectious titer); toxicity (resazurin reduction, ATP quantification)	Chaput et al., 2025 [92]

I = immortalized; P = Primary; BSL-2: biosafety level 2 laboratory; GFP: green fluorescent protein; Luc: luciferase; MOA: mechanism of action; PTV: Punta Toro virus; RVFV: Rift Valley fever virus; VLP: virus like particles; VSV: vesicular stomatitis virus.

**Table 2:** Animal models used for antiviral development or pathogenesis studies on RVFV.

Species	References	Strain	Sex	Age ^a^/Weight (g)	Exposure Route ^b^	Survival Rate ^c^	Clinical and Physiopathological Features ^d^	Targeted Organs, Tissues and Cells
Mice BALB/c	Smith et al., 2010 [44]	ZH501	Female	6–8 w	SC (10^3^ pfu)	0% (3–9 dpi)	Hepatitis followed by encephalitis Increase in liver enzymes (ALT, ALP) and bilirubin, RBC, hemoglobin, eosinophils, basophils, hematocrit Decrease in albumin, lymphocytes, neutrophils	Blood, liver, spleen, brain, kidney, heart, adrenal glands, ovaries, lungs, eyes Cells: epithelial, mesenchymal, neural, hematopoietic, endocrine. Mainly hepatocytes, liver macrophages, follicular dendritic cells in the spleen, neurons in the brain
	Reed et al., 2013 [93]	ZH501	Female	6–8 w	Aerosol or SC (10^3^ pfu)	0% (3–10 dpi)		
	Allen et al., 2018 [94]	ZH501	Female	6–8 w	SC (20 pfu)	0% (5–11 dpi)		
	Johnson et al., 2022 [62]	ZH501	Female	6–8 w	IP (100 pfu)	0% (3–9 dpi)		
	Gutjahr et al., 2020 [66]	35/74	Male and Female	3–6 m	IP (100 TCID_50_)	17% (3–8 dpi)		
	Wichgers Schreur et al., 2020 [95]	35/74	Female	6 w	IP (10^3^ TCID_50_)	0% (2–3 dpi)		
	Lacote et al., 2022 [96]	Smithburn and Clone 13	Female	6–8 w	IN (10^3^ TCID_50_)	40% (5–7 dpi) Smithburn; 35% (6–13 dpi) Clone 13	Encephalitis	Blood, brain, liver Cells: microglia, neurons
Mice C57BL6	Gray et al., 2012 [97]	ZH501	Female	8–10 w	SC (10^3^ pfu)	0% (2–4 dpi)	Hepatitis Decrease in glucose, lymphocytes, monocytes, RBC, platelets Increased eosinophils, ALT, bilirubin Strong inflammatory response with elevated cytokines in serum, liver, spleen, and brain (IL-6, KC, MCP-1, MIP-1a, G-CSF, IL-8, IFN-β, depending on tissues)	Blood, spleen, liver and brain Cells: hepatocytes
	Cartwright et al., 2020 [98]	ZH501	Male and Female	6–8 w	FP (2 TCID_50_)	0% (3–4 dpi)		
Mice CC057/Unc	Cartwright et al., 2022 [99]	ZH501	Male and Female	4–12 w	FP (2 TCID_50_)	0% (11–12 dpi)	Encephalitis Increase in neutrophils, platelets, hemoglobin (at 10–12 dpi) Decrease in WBC, lymphocytes and hemoglobin (at 3 dpi) Elevated cytokines and chemokines in the central nervous system (IL-10, IP-10, IL-6, MIG, MCP-1), and endothelial markers (ICAM-1, PAI-1, and thrombomodulin)	Brain, eye, spleen, blood, small intestine, liver, sciatic nerve, spinal cord Cells: hepatocytes
Mice 129S6SvEv STAT 1 knock-out	Lang et al., 2016 [100]; Lang et al., 2019 [101]	MP-12	Female	7 w	IN (1.6 × 10^6^ TCID_50_)	50% (6–10 dpi) (2016) 0% (6–8 dpi) (2019)	Hepatitis followed by encephalitis	Liver, spleen, brain
Mice 129/SvPasIco IFNAR −/−	Smee et al., 2018 [74]	MP-12	Male and Female	8–10 w	IP (40 pfu)	0% (4–8 dpi)	Hepatitis	Liver, serum, brain
Hamsters Syrian golden	Scharton et al., 2015 [102]	ZH501	Female	90–115 g	SC (1 or 10 pfu)	0% with 10 pfu (2–3 dpi)	Hepatitis followed by encephalitis (extended survival under ribavirin treatment) Increase in ALT	Blood, liver, spleen, lung, kidney, adrenal gland, brain, pancreas, intestine, eye
	Scharton et al., 2014 [81]	ZH501	Female	90–115 g	SC (30 pfu)	0% (2–3 dpi)		
	Westover et al., 2018 [73]	ZH501	Female	81–90 g	SC (30 pfu)	0% (2–4 dpi)		
Hamsters Syrian golden STAT 2 knock-out	Hickerson et al., 2018 [103]	MP-12	Male and female	7–8 w	Aerosol (150 pfu)	0% (5–7 dpi)	Hepatitis Increase in ALT and AST, neutrophils Decrease in cholesterol, platelets, lymphocytes	Liver, spleen, kidney, lung, brain, heart, serum, intestine
Domestic ferrets	Barbeau et al., 2020 [104]	ZH501	Male	6–9 m (1.1–1.9 g)	IN (10^6^ TCID_50_) ID (10^6^ TCID_50_)	25% (7–10 dpi) 100%	Encephalitis Decrease in lymphocytes and albumin levels Increase in neutrophils, total protein, liver enzymes (AST and ALT for IN exposure; ALT only for ID)	Blood, brain, spleen, lungs, eye
Gerbils Tum:(MON)	Anderson et al., 1988 [105]	ZH501	Female	3–64 w	SC (10^7^ pfu)	0% (3 w, MTD 6.3 dpi) 7% (5 w, MTD 7.8 dpi) 90% (7 w, MTD 9.5 dpi)	Encephalitis	Serum, spleen, brain (4 w)
Rats Wistar-Furth	Bales et al., 2012 [106]	ZH501	Female	8–10 w	Aerosol (0.4 and 240 pfu)	0% (3–12 and 3–6 dpi respectively)	Hepatitis followed by encephalitis if surviving hepatitis	Blood, liver, spleen, lung, heart, kidney, brain
	Caroline et al., 2014 [107]	ZH501	Female	8–10 w	Aerosol (50 pfu)	0% (4–6 dpi)		
	Peters and Slone, 1982 [108]	ZH501	Female	10–15 w	SC (10^3.7^ and 10^5.7^ pfu)	0 and 10% (MTD 3 and 3.2 dpi)		
Rats August Copenhagen Irish	Bales et al., 2012 [106]	ZH501	Female	8–10 w	Aerosol (0.4 to 3900 pfu)	0% with 3900 pfu (6–8 dpi)	Encephalitis	Brain
	Peters and Slone, 1982 [108]	ZH501	Female	10–15 w	SC (10^3.7^ and 10^5.7^ pfu)	50 and 90% (MTD 15 and 16 dpi)		
Rats Lewis	Bales et al., 2012 [106]	ZH501	Female	8–10 w	Aerosol (1.5 to 4400 pfu)	0% with 4400 pfu (6–9 dpi)	Encephalitis Decrease in lymphocytes, platelets, granulocytes Elevated chemokine and cytokine in serum and brain (MCP-1, M-CSF, Gro/KC, RANTES, IL-1β, IL-18, IL-1α, EPO, IFN-γ, VEGF, MIP-3α, IL-10); IL-13 decrease transiently	Blood, brain (olfactory bulb, cortex, cerebellum, brain stem, spinal cord), eye, cervical lymph node, salivary glands, liver, spleen Cells: neurons
	Caroline et al., 2016 [45]	ZH501	Female	8–10 w	Aerosol (25,000 pfu)	Not assessed		
	Walter et al., 2019 [109]	ZH501	Female	8–10 w	Aerosol (10^3^ pfu)	0% (6–7 dpi)		
	Peters and Slone, 1982 [108]	ZH501	Female	10–15 w	SC (10^3.7^ and 10^5.7^ pfu)	100%		
Rats Maxx	Peters and Slone, 1982 [108]	ZH501	Female	10–15 w	SC (10^3.7^ and 10^5.7^ pfu)	60 and 50% (MTD 11 and 13.8 dpi)	Encephalitis	Brain
Rats Sprague Dawley	Schwarz et al., 2022 [110]	ZH501	NI	8–10 w	SC (10^3^ pfu)	30% (2–4 dpi)	Ocular form, hepatitis Elevated chemokine and proinflammatory cytokines in eye (GM-CSF, GRO/KC, MCP-1, MIP-1α, IL-1β)	Eye (uvea, ciliary body, retina, optic nerve), liver, brain
Monkeys Rhesus macaques	Morrill et al., 1990 [111] (also in Morrill et al., 1989 [112] as controls)	ZH501	Male and female	Adult	IV (10^5^ pfu)	82% (6–15 dpi)	Hepatitis/hemorrhagic fever and encephalitis Increase in AST, ALT, gamma-glutamyl transferase, creatine kinase, interferon levels Decrease in hematocrit, platelet, lymphocyte and neutrophile counts	Liver, spleen, serum, adrenal glands, kidneys
	Smith et al., 2012 [113]	ZH501	Male and female	Adult (3–4 y)	IV, SC, IN 10^7^ pfu	100%	No clinical illness Increase in ALT Variation in WBC but no clear trend	Blood
	Hartman et al., 2014 [114]	ZH501	Female and male	Adult	Aerosolized (10^5^ pfu)	100%	No clinical illness, only temperature variations	NI
Monkeys Common marmoset	Smith et al., 2012 [113]	ZH501	Male and female	Adult (2–11 y)	IV, SC, IN 10^7^ pfu	75% (IV, 2 dpi) 50% (SC, 4–12 dpi) 100% (IN, 8–11 dpi)	IV exposure: hepatitis Increase in ALT levels Variation in WBC but no clear trend SC exposure: hepatitis followed by encephalitis Increase in ALT and ALP, BUN, creatinine IN exposure: encephalitis Increase in ALT and ALP, BUN and creatinine Decrease in WBC	Liver, spleen, brain, serum, adrenal gland, kidney, lung, heart, lymph nodes, intestines, gonads, skeletal muscle, bone marrow, eye Cells: hepatocytes (IV exposure) and neurons (SC exposure)
Monkeys Common marmoset	Hartman et al., 2014 [114]	ZH501	Female and male	Adult	Aerosolized (10^1^–10^5^ pfu)	50% (depending on dose, 9–10 dpi, estimated LD_50_ = 3.5 × 10^3^ pfu)	Encephalitis Increase in WBC (granulocytes), platelet volume and distribution, ALP and BUN Decrease in the number of platelets Clotting times slightly elevated	Brain, eye, kidney, liver, lung, sciatic nerve, spinal cord, spleen Cells: neurons
Monkey African green monkey	Hartman et al., 2014 [114]	ZH501	Female and male	Adult	Aerosolized (10^5^ pfu)	17% (8–11 dpi)	Encephalitis Increase in lymphocyte and granulocyte counts, BUN, glucose Elevated clotting times	Brain, spinal cord, eye, spleen Cells: neurons
Monkey Cynomolgous macaque	Hartman et al., 2014 [114]	ZH501	Female and male	Adult	Aerosolized (10^5^ pfu)	100%	Mild form	-

^a^ w: weeks, m: months, and y: years. ^b^ SC: subcutaneous, IP: intraperitoneal, FP: footpad, IN: intranasal, ID: intradermal, and IV: intravenous; pfu: plaque-forming units and TCID50: tissue culture infectious dose 50%. ^c^ dpi: day post-infection, MTD: mean time of death. ^d^ ALT: alanine aminotransferase, ALP: alkaline phosphatase, AST: aspartate aminotransferase, RBC: red blood cell, and WBC: white blood cell.

**Table 3:** In vitro antiviral studies against CCHFV or surrogate HAZV.

Virus (Strain)	Specificity	Cell Line (Type and Origin)	Assays (Readout)	References
CCHFV (IbAr10200 wild-type, with ZsGreen (S-segment) or mCherry (M-segment) reporters) VSV-GFP (CCHFV-GPC)	Reporter virus (fluorescent: ZsGreen or mCherry) and pseudovirus expressing a fluorescent protein	BHK-21 (Baby hamster kidney; I); SW-13 (Human adrenocortical carcinoma cell; I); Huh7.5 (Human liver epithelial cells; I)	Screening, dose response, and MOA (plaque reduction neutralization assay and fluorescence); toxicity (MTT reduction)	Mears et al., 2022 [186]
CCHFV (IbAr10200 wild-type and ΔS-ZsGreen)	Reporter virus (fluorescent)	SW-13 (Human adrenocortical carcinoma cell; I); Huh7 (Human liver epithelial cells; I)	Dose response (fluorescence and infectious titer); toxicity (ATP quantification)	Welch et al., 2017 [187]
CCHFV (Kosova Hoti minigenome) HAZV (JC280 wild-type and minigenome ΔS-Luc)	Minigenome expressing a luminescent protein BSL-2 surrogate expressing a luminescent protein	BHK-21 (Baby hamster kidney; I); SW-13 (Human adrenocortical carcinoma cell; I)	Screening, dose response, and MOA (luciferase and infectious titer); toxicity (resazurin reduction)	Hirano et al., 2022 [188]
HAZV (JC280) CCHFV (IbAr 10200)	BSL-2 surrogate	Vero (African green monkey epithelial kidney cells; I)	Dose response and MOA (virus titer: immunofluorescence (protein N)); toxicity (MTT reduction)	Mirandola et al., 2021 [189]
CCHFV (Afg09-2990)	-	Primary murine hepatocytes (P)	Dose response (infectious titer); toxicity (MTT reduction)	Müller et al., 2020 [190]
CCHFV (YL16070)	-	VeroE6 (African green monkey epithelial kidney cells; I)	Screening, dose response, and MOA (virus titer: immunofluorescence (protein N)); toxicity (WST-8 reduction)	Wang et al., 2022 [191]
VSV-Luc (CCHFV-GPC-Luc with GPC from IbAr10200, Sudan Al-Fulah 200903, Turkey-200406546, Kosova Hoti, Oman-199809166, SPU18/88, ArD15786, YL04057, Afg09, NIV112143, and Baghdad-12) CCHFV (wild-type Turkey-812955, Oman-812956, UAE-813040, UAE-813042, and IbAr 10200)	Pseudoviruses of various strains expressing a luminescent protein	SW-13 (Human adrenocortical carcinoma cell; I)	Screening and dose response (luciferase, infectious titers, plaque reduction neutralization assay); toxicity (MTT reduction)	Zivcec et al., 2017, 2015 [183,192]
CCHFV (IbAr 10200, Hy-13, UG3010, and Spu128/81)	-	SW-13 (Human adrenocortical carcinoma cell; I)	Dose response and MOA (cell staining); toxicity (cell staining)	Paragas et al., 2003 [193]
CCHFV (Kosova Hoti)	-	A549 (Human lung epithelial cells; I)	Dose response (viral RNA quantification); toxicity (cell staining and ATP quantification)	Földes et al., 2020 [194]
CCHFV (IbAr10200 and ArD39554)	-	VeroE6 (African green monkey epithelial kidney cells; I); Huh7 (Human liver epithelial cells; I)	Dose response and MOA (viral titer); toxicity (MTT reduction)	Ferraris et al., 2015 [195]
CCHFV (Afg09-2990)	-	VeroE6 (African green monkey epithelial kidney cells; I)	Dose response (virus titer: immunofluorescence (protein N)); toxicity (MTT reduction	Oestereich et al., 2014 [196]
HAZV (JC280)	BSL-2 surrogate	A549 (Human lung epithelial cells; I)	Dose response and MOA (Virus titer: immunofluorescence with mouse hyperimmune ascitic fluid)	Flusin et al., 2011 [197]
CCHFV (IbAr10200-eGFP and minigenome)	Reporter virus (fluorescent) Minigenome expressing a fluorescent protein	HUVEC (Human umbilical vein endothelial cells; P)	Dose response and MOA (fluorescence, viral RNA quantification); toxicity (WST-8 reduction)	Liu et al., 2024 [198]
CCHFV (YL16070)	-	HEK 293 (Human embryonic kidney cells; I)	Dose response (Viral RNA and protein N quantification, luciferase)	Du et al. 2025 [199]

I = immortalized; P = primary; BSL-2: biosafety level 2 laboratory; CCHFV: Crimean-Congo hemorrhagic fever; GFP: green fluorescent protein; GPC: polyprotein precursor; HAZV: Hazara virus; Luc: luciferase; MOA: mechanism of action; VSV: vesicular stomatitis virus.

**Table 4:** Animal models used for antiviral development or pathogenesis studies on CCHFV.

Species	References	Strain	Sex	Age ^a^/Weight (g)	Exposure Route ^b^	Survival Rate ^c^	Clinical and Physiopathological Features ^d^	Targeted Organs, Tissues and Cells
Mice C57BL6 IFNAR −/−	Zivcec et al., 2013 [172]	IbAr 10200	Male and Female	6–12 w	SC (10 TCID_50_)	0% (4–7 dpi)	Hepatitis Weight loss, ruffled fur, hunched posture, lethargy Increase in liver enzymes (ALT, AST), globulin, total protein, sodium, potassium, mean platelet volume, blood clotting time Decrease in blood glucose, albumin, platelet count, fibrinogen Strong proinflammatory immune response (G-CSF, IFN-γ, CXCL10, CCL2, GM-CSF, IL-1α, IL-1β, IL-2, IL-6, IL-12p70, IL-13, IL-17, CXCL1, CCL3, CCL5, and TNF-α significatively elevated in serum)	Blood, liver, spleen, kidney, lungs, brain, eye, lymph node Cells: endothelial and phagocytes Mainly hepatocytes, Kupffer cells and macrophages in the liver; macrophages in the lymph nodes and spleen Liver and spleen damage
	Hawman et al., 2018 * [200]	Kosova Hoti		12–18 w	IP (5 TCID_50_)	0% (8 dpi)		
	Wang et al., 2022 * [191]	YL16070		12–18 w	IP (3000 or 5000 TCID_50_)	20% (5–7 dpi) or 14% (4–6 dpi)		
	Liu et al., 2024 * [198]	YL16070	Female	7–12 w	IP (3000 TCID_50_)	16.7–33% (5–7 dpi)		
	Fels et al., 2021 * [201]	IbAr 10200	Male and Female	5–8 w	IP (100 pfu)	5% (3–5 dpi)		
	Garrison et al., 2024 * [202]	IbAr 10200	Female	7–9 w	SC (100 pfu)	0% (4–8 dpi)		
	Golden et al., 2019 * [203]	IbAr 10200	Female	6–15 w	SC (100 pfu)	0% (4–5 dpi)		
Mice C57BL6J Antibody-mediated IFN-I blockade	Lindquist et al. 2018 [204]	Afg 09-2990	NI	6–8 w	IP (100 pfu)	0% (5 dpi)	Weight loss, hunched posture, rough coat, hypoactivity Elevated inflammatory cytokines and chemokines in blood (CCL2, CCL4, CXCL1, CXCL10, IFN-γ, IL-1β, IL-6, IL-18, GM-CSF and TNF-α) Increase in liver enzymes (ALT, AST)	Blood, liver, and spleen Cells: mainly hepatocytes, Kupffer cells and macrophages in the liver Liver damage
	Sorvillo et al., 2024 [205]	IbAr 10200	Male and Female	6–83 w (independent of age)	SC (100 pfu)	1% (4–8 dpi) with		
Mice 129Sv IFNAR −/−	Bereczky et al., 2010 [171]	IbAr 10200	Female	7–10 w	IP (10^1^, 10^2^, 10^5^ and 10^6^ pfu)	0% (2–4 dpi)	Hepatitis Weight loss, labored breathing, porphyry around the nostrils and eyes Increase in liver enzymes (ALT, AST)	Blood, liver, spleen, lungs, kidney, brain, heart Cells: Mainly hepatocytes, Kupffer cells Liver and spleen damage
	Oestereich et al., 2014 * [196]	Afg09-2990	Female	6–12 w	IP (100 pfu)	0% (3–6 dpi)		
	Kempster et al., 2024 * [206]	IbAr 10200	Male and Female	5–8 w	ID (10 ffu)	0% (5 dpi)		
Mice 129 S6/Sv STAT1 −/−	Bente et al., 2010 * [170]	IbAr 10200	Male and Female	3–6 w	IP (100 pfu)	0% (3–5 dpi)	Weight loss, lethargy, piloerection, hunched posture, fever on 2 dpi then hypothermia Decrease in WBC and platelet counts Increase in liver enzyme (ALT) Elevated inflammatory cytokines and chemokines in blood (IFN-α, IFN-β, CCL2, IFN-γ, IL-1β, IL-6, IL-10, and TNF-α).	Blood, liver, spleen, lung, kidney, brain Cells: Mainly hepatocytes, Kupffer cells, occasionally liver endothelial cells Liver and spleen damage
	Fels et al., 2021 * [201]	Turkey 2004	Female	4–8 w	IP (100 pfu)	0% (5–7 dpi)		
Mice C57BL6J	Hawman et al., 2021 [156]	MA-CCHFV (Mouse adaptated strain from Kosova Hoti)	Male	8 w	IP (10^4^ TCID_50_)	93% (NI)	Hepatitis Weight loss, piloerection, hunched posture, lethargy Increase in levels of liver enzymes (ALT, AST). Elevated inflammatory cytokines and chemokines in blood (IFN-α, IFN-β, IFN-γ, CCL2, CCL3, CCL4, CCL5, CCL11, IL-1β, IL-5, IL-6, IL-10, G-CSF, TNF-α and CXCL1)	Blood, liver, spleen, lung, kidney, brain. Cells: Mainly hepatocytes, Kupffer cells, liver endothelial cells Liver and spleen damage
	Tipih et al., 2023 * [207]			6–8 w	IP (10^5^ TCID_50_)	17% (5–6 dpi)		
Humanized mice Hu-NSG™-SGM3	Spengler et al., 2017 [208]	Oman-199809166 Turkey-200406546	Female	NI (16 w after engraftment)	IP (10^4^ TCID_50_)	100% (Oman strain) 0% (Turkey strain; 12–23 dpi)	Weight loss	Blood, brain, liver, spleen, eye, kidney, adrenal glands, ovary Cells: histiocytes and multinucleated giant macrophages; Kupffer and endothelial cells; rare hepatocytes; astrocytes, rare glial cells and neurons Liver, spleen, brain, and lung damage
Hamsters Syrian golden STAT2 −/−	Ranadheera et al., 2020 * [209]	IbAr 10200	Female	6–8 w	SC (100 TCID_50_)	0% (5–12 dpi)	Weight loss, hunched posture, ruffled and dull coat condition, aggressive behavior, severe dehydration, lethargy, labored breathing, disorientation, jerky movements, righting reflex issues, hind limb paralysis, ocular, anal, or petechial hemorrhaging. Decrease in platelets on 6 dpi, then significatively higher than uninfected on 10 dpi Decrease in albumin and total proteins Increase in WBC, globulin, ALT, blood clotting time Elevated expression of β2M, TGF-β, p27, TNF-α, and IFNγ genes.	Blood, liver, spleen, lung, kidney, heart Cells: endothelial and epithelial Liver and spleen damage
Monkey Cynomolgus macaque	Haddock et al., 2018 [178]	Kosova Hoti	Female and male	3–5 y	IV + SC (10^5^ TCID_50_)	75% (6–7 dpi)	Piloerection, anorexia, hunched posture, facial and body edema, lethargy, epistaxis, hematochezia, gingival hemorrhage, petechiae, deep tissue hemorrhaging Decrease in WBC, platelets, total protein, albumin Increase in liver enzymes (AST and ALT), blood clotting time Elevated inflammatory cytokines and chemokines in blood (IL-6, IL-10, IL-15, IL-17a, IL-1RA, MCP-1, MIP-1β, IL-1β).	Blood, lymph nodes, liver, spleen, adrenal glands, kidney Positive oral and nasal swabs Cells: hepatocytes, Kupffer, and endothelial cells Liver and spleen damage
					IV (10^5^ TCID_50_)	25% (7 dpi)		
	Hawman et al., 2020 * [210]	Kosova Hoti	Female and male	Adult	IV + SC (10^5^ TCID_50_)	87.5% (1/8, 5 dpi)		
	Cross et al., 2020 [211]	SCT ex Afghanistan, Kosova Hoti	Female and male	3–8 y >18 y	IV (2.10^5^ pfu)	100%		
Monkey Rhesus macaque	Hawman et al., 2024 [212]	CMP-CCHFV (strain adapted in cynomolgus from strain Kosova Hoti)	Female and male	2.9–8.6 y	IV + SC (10^5^ TCID_50_)	100%	Decrease in platelets for 3/8 animals Increase in hepatic enzymes for 2/8 animal	Virus detected in the blood of all animals and measured only in the organs of the ones presenting elevated hepatic enzymes (detected in lymph nodes, spleen, liver, kidney, adrenal glands)

NI: not indicated; * studies with antiviral activity assessment. ^a^ w: weeks, and y: years. ^b^ SC: subcutaneous, IP: intraperitoneal, ID: intradermal, and IV: intravenous; pfu: plaque-forming units and TCID_50_: tissue culture infectious dose 50%. ^c^ dpi: day post-infection. ^d^ ALT: alanine aminotransferase, ALP: alkaline phosphatase, AST: aspartate aminotransferase, RBC: red blood cell, and WBC: white blood cell.

## Data Availability

No new data were created or analyzed in this study. Data sharing is not applicable to this article.

## Abbreviations

The following abbreviations are used in this manuscript:

AGM	African green monkey
ALP	Alkaline phosphatase
ALT	Alanine aminotransferase
AST	Aspartate aminotransferase
BSL	Biosafety laboratory level
CCHF	Crimean Congo hemorrhagic fever
CCHFV	Crimean Congo hemorrhagic fever virus
DIC	Disseminated intravascular coagulation
FP	Footpad
GFP	Green fluorescent protein
HAZV	Hazara virus
ID	Intradermal
IFN	Interferon
IN	Intranasal
IP	Intraperitoneal
IV	Intravenous
LDH	Lactate dehydrogenase
MLD	Mucin-like domain
NHP	Non-human primate
PTV	Punta Toro virus
RVFV	Rift Valley fever virus
SC	Subcutaneous
VLP	Virus-like particle
VSV	Vesicular stomatitis virus
WHO	World Health Organization
